# *Erratum:* Vol. 65, Nos. 50 & 51

**DOI:** 10.15585/mmwr.mm6601a10

**Published:** 2017-01-13

In the report “Increases in Drug and Opioid-Involved Overdose Deaths — United States, 2010–2015” in both “TABLE 1. Number and age-adjusted rate of drug overdose deaths* involving natural and semisynthetic opioids^†^ and methadone,^§,¶^ by sex, age group, race/ethnicity,** U.S. Census region, and selected states^††^ — United States, 2014 and 2015,” and in “TABLE 2. Number and age-adjusted rate of drug overdose deaths* involving synthetic opioids other than methadone^†^ and heroin,^§,¶^ by sex, age group, race/ethnicity,** U.S. Census region, and selected states^††^ — United States, 2014 and 2015,” the seventh footnote (^§§^) should have read as follows: “Statistically significant at p<0.05 level. **Nonoverlapping confidence intervals based on the gamma method **were used if the number of deaths was <100 in 2014 or 2015, and z-tests were used if the number of deaths was ≥100 in both 2014 and 2015.”

